# Subsequent Dyslipidemia and Factors Associated with Mortality in Schizophrenia: A Population-Based Study in Taiwan

**DOI:** 10.3390/healthcare9050545

**Published:** 2021-05-07

**Authors:** Mei-Chi Hsu, Wen-Chen Ouyang

**Affiliations:** 1 Department of Nursing, I-Shou University, Kaohsiung City 82445, Taiwan; hsu6889@gmail.com; 2 Department of Geriatric Psychiatry, Jianan Psychiatric Center, Ministry of Health and Welfare, Tainan City 71742, Taiwan; 3 Department of Nursing, Shu-Zen Junior College of Medicine and Management, Kaohsiung City 82144, Taiwan; 4 Department of Psychiatry, College of Medicine, Kaohsiung Medical University, Kaohsiung City 80708, Taiwan

**Keywords:** subsequent dyslipidemia, schizophrenia, mortality, U-shaped pattern, comorbidity, early detection

## Abstract

*Background:* Persons with schizophrenia are at greater risk of developing subsequent medical conditions. To date, few studies have examined comprehensively the risks, mortality and survival rates in schizophrenia and subsequent dyslipidemia over different time periods. The objective of this study was to evaluate the occurrence of subsequent dyslipidemia after the diagnosis of schizophrenia, and factors associated with mortality and survival rate in patients with schizophrenia. *Methods:* We used a population-based cohort from Taiwan National Health Insurance Research Database, to investigate in patients whom were first diagnosed with schizophrenia during the period from 1997 through 2009, the risk of subsequent dyslipidemia during follow-up. Cumulative incidences and hazard ratios after adjusting for competing mortality risks were calculated. *Results:* A total of 20,964 eligible patients were included. Risks (i.e., comorbidity) and protective factors (i.e., statin use) have significant impacts on mortality. The mortality exhibits a U-shaped pattern by age. After 50, the risk of death increases with age. Risk of mortality before 50 increases with a decrease in age. Risks differed by the duration time to subsequent dyslipidemia after schizophrenia. Mean duration was 63.55 months in the survive group, and 43.19 months in the deceased group. The 5-, 10-, and 15-year survival rates for patients with schizophrenia and subsequent dyslipidemia were 97.5, 90, and 79.18%, respectively. *Conclusion:* Early occurrence of subsequent dyslipidemia is associated with increased overall mortality in patients with schizophrenia.

## 1. Introduction 

Evidence suggests that persons with schizophrenia are at greater risk of developing the subsequent endocrine and metabolic disease [1]. Indeed, metabolic syndrome, a cause of the increasing comorbidity, is a major concern for persons with schizophrenia who have a 4-fold risk in comparison with the general population [2,3,4,5]. Epidemiological studies have shown that persons with schizophrenia have higher mortality rates and shorter life span. It is also known that metabolic syndrome is associated with a 1.5-fold increase in all causes of death [6,7]. In schizophrenia, the main cause of natural death is due to cardiovascular disease. One of the risk factors of increased death of cardiovascular disease is dyslipidemia—an imbalanced level of blood lipid component. Dyslipidemia is one of the most prevalent metabolic syndromes in persons with schizophrenia. Approximately 17% of patients with schizophrenia developed dyslipidemia. Development of dyslipidemia in schizophrenia is associated with advanced age, cognitive impairment, lifestyle patterns, poor diet, poverty, the treatment of schizophrenia (antipsychotic medications), and imbalanced blood lipid components. The presence of dyslipidemia causes significant risk to heart disease, stroke and other cardiovascular-related morbidity, and mortality [8]. The relationships between schizophrenia and subsequent dyslipidemia (dyslipidemia after the diagnosis of schizophrenia) play a crucial role in clinical management of both schizophrenia and cardiovascular disease.

Pathophysiology of the disease is rather intricate, involving the interaction of many factors, such as genetic, environmental, and immunological. A recent study [1] explored the associations between 10 broad types of mental disorders and 9 subsequent broad categories of comorbidities. This study provides robust and comprehensive findings to support the associations between mental disorders, including schizophrenia, and subsequent medical conditions. These physical and medical conditions are associated with numerous negative outcomes, such as increased morbidity and mortality. Recent studies have shown a greater increase in mortality for schizophrenia and chronic psychoses [9,10]. The risk of mortality is 2–3 times higher in patients with schizophrenia than that in the general population [11]. Persons with schizophrenia or chronic psychoses have an average life expectancy of 64.7 years [9], which is a 20% shorter life expectancy in comparison with general population [12]. More than 60% of this premature mortality are due to comorbid somatic conditions, including dyslipidemia [5,9,13].

The lipids disfunctions may also worsen psychoses on a psychopathological point of view, for example increasing suicide risk. Apart from schizophrenia, evidence suggests an association between metabolic factors (i.e., lipid profile) and suicidality in subjects with bipolar disorder or depression [14,15]. A study of Orsolini et al. [14] has indicated the associations between abnormal lipid contents in peripheral tissues and suicide. Dysregulation of lipid levels tended to be apparent in suicidal depressive patients compared with non-suicidal patients [14]. C-reactive protein and total cholesterol may be altered in patients with bipolar I disorder [15].

The prevalence of hypertriglyceridemia and low levels of high-density lipoprotein increased substantially in persons with schizophrenia (35.2% and 42.6%, respectively) compared to the healthy control [16]. Current advances in studies of applied lipidomics and analysis have particularly focused on multifaceted role of lipids in schizophrenia, and changes of lipid profiles in relation to different treatment response. For example, persons whose total cholesterol levels >200 mg/dL are found to be correlated with lack of response to fluoxetine and nortriptyline [17]. Disturbed lipid metabolism and signaling drives changes associated with neurophysiological activity and etiology in schizophrenia. Furthermore, abnormal serum lipids are biological factors strongly associated with cognitive dysfunction, suicide attempts, and violent behavior [18,19,20].

Antipsychotics are generally the first line and cornerstone treatment for schizophrenia. However, antipsychotics, particularly the second-generation antipsychotics, can also pose a substantial risk for developing metabolic instability [9,21]. Antipsychotic-induced dyslipidemia would increase risk for cardiovascular diseases or premature cardiovascular mortality.

Prevention and treatment of dyslipidemia requires early diagnosis and effective lipid monitoring. Unfortunately, regular lipid monitoring is often less accessible for the psychiatric populations [22,23]. Occurrence of dyslipidemia in patients with schizophrenia is often neglected or disregarded by both mental health professionals and patients themselves. This accompanied by suboptimal physical health care in these patients may have accounted for the high prevalence of cardiovascular disease and mortality [8,24]. Early detection of dyslipidemia permits opportunity to identify the underlying medical cause and implements interventions against predisposing and precipitating risk factors. Hence, early diagnosis and treatment is an effective way to minimize the impact of dyslipidemia; in other words, prevention and treatment of dyslipidemia requires early diagnosis and effective lipid monitoring.

Detection and recognition of dyslipidemia in patients with schizophrenia is also important for mental health professionals, because of their critical role in the management and care of patients with both schizophrenia and dyslipidemia. A clear addressed strategy to improve recognition of subsequent dyslipidemia can reduce many adverse outcomes for this vulnerable population.

In Taiwan, the prevalence rate of dyslipidemia has increased significantly in recent years. Even though the mechanism and clinical guidelines of disease treatment have been well established in Taiwan [25,26], guidance for the role of mental health professionals in the care of patients with severe mental disorders and dyslipidemia are lacking in the guidelines.

To ensure that quality of care is maintained at desired outcomes, psychiatric healthcare services require continuous improvement. There is no doubt that the challenge of managing the care of patients with both schizophrenia and dyslipidemia is enormous. Appropriate management of dyslipidemia, especially in patients with a family history of heart disease, can significantly reduce the risk for heart disease, and improve rate of survival.

To date, few studies have provided comprehensive data regarding risks, mortality and survival rates of schizophrenia and subsequent dyslipidemia over a particular time period. The association between subsequent dyslipidemia after the diagnosis of schizophrenia, and mortality and survival rates in patients with schizophrenia also remains to be clarified. The aim of this study was to evaluate the occurrence of subsequent dyslipidemia after the diagnosis of schizophrenia, and factors associated with mortality and survival rate in patients with schizophrenia. The role of mental health professionals in supporting patients and promoting disease understanding, patient assessment and education and treatment adherence is also provided.

## 2. Materials and Methods

### 2.1. Data Sources and Study Population

The nationwide population-based retrospective cohort study design was used. Data were retrieved from Taiwan National Health Insurance Research Database (NHIRD). The NHIRD includes very detailed claim data of all forms of healthcare service for reimbursement and registration files from 99.9% of the 23 million inhabitants of Taiwan since 1995. Demographic data, dates of clinical visits, diagnostic codes, complete prescription details, expenditure amounts, and others are all included in the database. International Classification of Diseases, Ninth Revision (ICD-9), were used to define diseases during the study period. Therefore, the selection bias of this study was minimal.

All patients included in this study, were ≥18 years, had a diagnosis of dyslipidemia occurred after the diagnosis of schizophrenia between 1 January 1997, and 31 December 2009, including a span of 13 years of exposure with drug prescriptions, admissions, and outpatient visit. Health data were collected from NHIRD. The date on which a patient with schizophrenia received was set as the index date.

**Figure 1:**
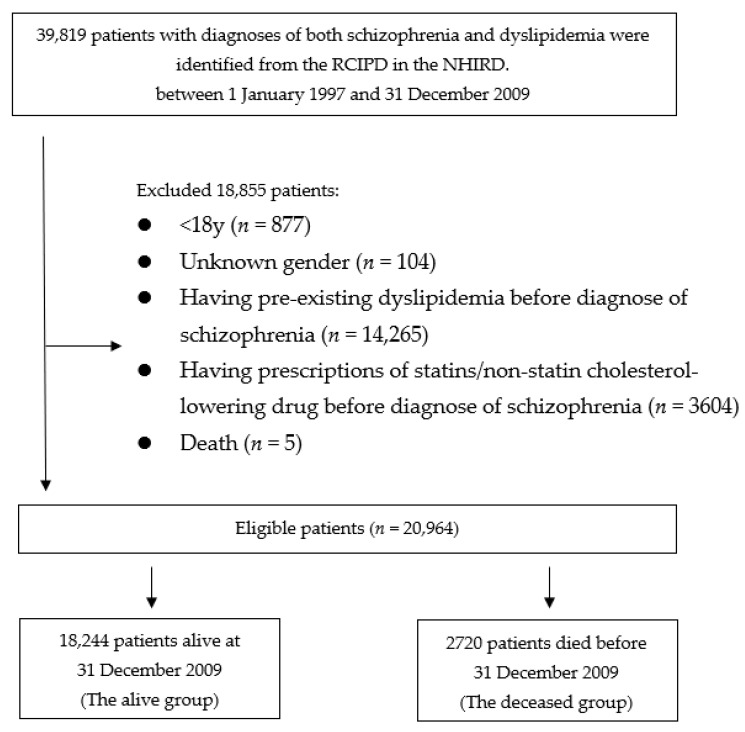
Flowchart of identification and enrolment of the study subjects.

One cohort was identified: patients who had schizophrenia and dyslipidemia. Then, they were divided into 2 groups according to live status: “alive” or “deceased”. Patients who died during the observation period, were defined as the deceased group, while those who were still alive after 31 December 2011, were defined as the alive group. Figure 1 presents a flowchart of identification and enrolment of the study subjects.

### 2.2. Surveillance for Dyslipidemia Development

To ensure diagnostic accuracy and validity as well as patient homogeneity, we identified study subjects diagnosed with subsequent dyslipidemia combined with concomitant drug treatment and had either outpatient or inpatient care in schizophrenia. Time to subsequent dyslipidemia was defined as duration from first hospital diagnosis of schizophrenia to time of subsequent dyslipidemia. Surveillance for dyslipidemia development continued until the time of death, or 31 December 2011, which represents a 2-year follow-up. Patients with history of pre-existing diagnoses of dyslipidemia prior to schizophrenia diagnosis, or the index date or whose diagnosis and death were the same day, or during the 6 months following the index date, were not included. Those who had received previous dyslipidemia treatment before the diagnosis of schizophrenia were also not included. Those patients withdrawn from the NHI claim system were excluded in the patient selection process. The purpose of this exclusion was to confirm the potential influence on patients with schizophrenia was related to the occurrence of dyslipidemia.

### 2.3. Concomitant Drug Therapy of Dyslipidemia and Comorbidities

The drug users were individuals who had received either statin and/or non-statin cholesterol-lowering drug throughout the observation period. The data retrieved included the claims data, medical orders, basic data files, and drug files. The major statins of interest comprised six different subtypes with ATC codes: simvastatin (C10AA01), lovastatin (C10AA02), atorvastatin (C10AA05), fluvastatin (C10AA04), pravastatin (C10AA03), and rosuvastatin (C10AA07).

Only diagnosis of the comorbidities on admission (from 2 years before the index date to 2 years following the index date) was included. The comorbidities (ICD-9 code #) include: malignant tumor (104–208), diabetes mellitus (ICD-9 code, 250), alcohol-related disease (291, 303, 305, 571), hypertension (401–405), acute coronary syndrome (410–414), heart failure (428), cerebrovascular accident (430–438), chronic obstructive pulmonary disease (COPD, 490–496), hepatic failure (570), liver diseases (e.g., cirrhosis) (571.2, 571.5), pancreatitis (577), and renal failure (584–586).

### 2.4. Overall Mortality Measurement

Death was defined as patient deceased and has withdrawn from the National Health Insurance claim system. The cause of death was determined based on the primary diagnosis at hospital admission during the last 3 months before death.

### 2.5. Ethical Considerations

The study was carried out in conformity with the principles set in the Declaration of Helsinki. The research protocol (Institutional Review Board, IRB No.: 15-038, 15-039) of this study was approved prior to implementation by the Institutional Review Board of the participated hospital. Use of database for research purpose was granted from the National Health Research Institute. Database included detailed medical records, such as outpatient, emergency, and hospitalization, and also principal or additional diagnosis codes and prescription. Patient consent was waived due to a database-based study by the IRB of Jianan Psychiatric Center. All personal information is encrypted for privacy protection.

### 2.6. Statistical Analysis

Precision and accuracy of the statistical information retrieved from database was ratified by 2 statisticians. Multivariable analyses and hazard ratios (HRs) using the Cox Proportional Hazard Regression Model (Cox Model) were adopted and analyzed for determination of the independent risk factors for mortality. Competing risk events, such as age, therapeutic agents, and comorbidities were stratified. The survival curve was analyzed using the Kaplan-Meier estimate. Comparisons of survival curves of two groups were computed using the log rank test. Person-years were calculated. Significance of differences between continuous variables were tested by using *t*-test, and *χ*2 tests were used to test relationships between categorical variables. Statistical analyses and modeling were done using SAS 9.4 software (SAS Institute, Inc. Cary, NC, USA). In this study, to compare the 95% confidence level (CI) for two-tailed tests, the significance level was set at 0.05.

## 3. Results

### 3.1. Study Population

Eligible patients were first identified (*n* = 39,819). We excluded 877 patients for being younger than 18 years old, and 104 patients with unknown gender. The remaining 38,838 patients were scrutinized for eligibility. A total of 3604 patients who received prescriptions of cholesterol-lowering drug prior to the diagnosis of schizophrenia, 14,265 patients who had previous diagnosis of dyslipidemia prior to schizophrenia, and 5 patients died less than 3 months of the follow-up were also excluded. Therefore, the remained 20,964 patients who were considered eligible were enrolled into the study cohorts. A total of 18,244 patients who were alive by 31 December 2009, were grouped into the alive group. The dead group comprised 2720 patients who died before 31 December 2009.

A total of 1063 and 133 patients aged ≥60 years, 1937 and 255 patients aged 50–59 years, and 15,244 and 2332 patients aged <50 years were included in alive and deceased groups, respectively (Table 1). The time for patients receiving dyslipidemia diagnosis after the diagnosis of schizophrenia was significantly delayed in the alive group than the deceased group. The mean (±standard deviation) overall observation person-year in the alive and deceased groups was 11.13 (±2.96) and 8.32 (±3.34) years, respectively. Patients in the deceased cohort were slightly younger with a mean age of 37.04, as compared to 37.55 in the alive cohort (*p* = 0.049).

The durations of months (mean ± SD) of dyslipidemia after patients receiving the diagnosis of schizophrenia (length of time between diagnosis of schizophrenia and diagnosis of dyslipidemia) for the alive cohort were 63.55 (±41.09) months, and for the deceased cohort, were 43.19 (±33.75) months. The duration to dyslipidemia diagnosis for patients in the alive cohort were significantly longer than that in the deceased cohort.

The incidence rates of comorbidities were higher in older patients than younger patients. The prevalence of comorbidities in the deceased group was significantly higher than that in the alive group (*p* < 0.0001). Hypertension was the most frequent comorbidity (>30%) in the deceased group. Renal failure was also more than 20%.

For age-based comparison, an age-specific trend of mortality was found. There is an age-specific U-shaped mortality pattern. Patients died at much younger and much older ages. Mortality rates start to increase beyond age 50, particularly at the oldest ages. Mortality is also high among much younger adults, after which it declines, reaching its lower level around ages 50. More than two-fifths of patients died after 15 years of follow-up.

Patients who received statin treatments include more than one type of statins. The highest prescribed statin was lovastatin in both groups (41.38% and 48.46%, respectively). The rates of use were significantly different between two groups (*p* < 0.0001).

### 3.2. Analysis of Overall Mortality Using Simple Cox Proportional Hazard Model

Results from the model show that receiving prescription of statin is protective in reducing the risk of death (Table 2), particularly for patients who were much younger or older. Treatment with statins was significantly associated with a reduced overall mortality as shown by a HR of 0.74 (*p* < 0.0001) for simvastatin, and 0.82 (*p* = 0.004) for fluvastatin. The risks for mortality were significantly higher in renal failure, followed by heart failure. Later onset of subsequent dyslipidemia has protective effects against mortality (HR = 0.99, 95% CI (0.99 to 0.99), *p* < 0.0001). When schizophrenia, dyslipidemia, and comorbidities occurred concurrently, liver disease and alcohol-related disease exerted a synergistic effect on risk of mortality.

### 3.3. Multivariable Analysis

The interaction between schizophrenia, dyslipidemia, and comorbidities in patients showed HRs ranging between 1.16 and 2.18 (Table 3). When schizophrenia, dyslipidemia, and alcohol-related disease occurred concurrently, the HR for mortality was 2.12 (95% CI (1.81–2.49), *p* < 0.0001). When schizophrenia, dyslipidemia, and renal failure occurred concurrently, the HR for mortality increased to 2.18 (95% CI (1.76–2.71), *p* < 0.0001). Heart failure was also a crucial comorbidity associated with mortality. Furthermore, later onset of HLD after schizophrenia also has protective effects against mortality (HR = 0.99, 95% CI (0.99 to 0.99), *p* < 0.0001).

Types of statins prescribed are also significantly associated with mortality (Table 3). Simvastatin use with an adjusted HR of 0.89 (95% CI (0.80 to 1), *p* = 0.042) was an independent protective factor for overall mortality and was significantly associated with lower risk of mortality.

### 3.4. Survival Rate

**Figure 2:**
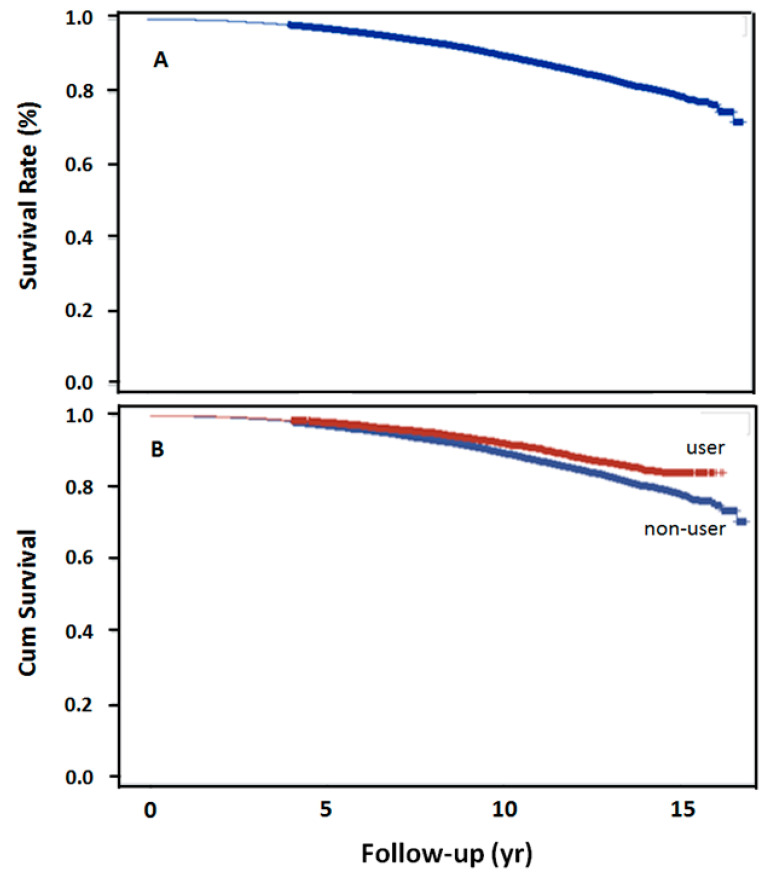
15-year overall survival rates in 2 groups. (**A**) Overall survival rate following schizophrenia and subsequent dyslipidemia (*n* = 18,244), (**B**) overall survival rate with the use of simvastatin following schizophrenia and dyslipidemia (*p* < 0.0001).

The log-rank test revealed the trend over the entire K–M curve (Figure 2A). The 5-, 10-, 15-year survival rates after dyslipidemia were 97.5%, 90%, and 79.18%, respectively. Survival rate was also significantly higher in the statin user than in the nonuser group, at 10-year (*p* < 0.001, Figure 2B).

## 4. Discussion

The novelty of this study is the use of a model to predict the possible impact on the clinical outcomes. We showed that specific patient-dependent risk and protective factors have long-term effect on mortality and survival. This study includes big data and health analytics, and provides data-driven insights to improve health outcomes. The present study has fully utilized all data during the course of 13 years of consecutive follow-up to detect event ascertainment and risk factors of dyslipidemia, mortality, and survival rate in schizophrenia. As comorbidities have a significant impact on survival, we have implemented in this study comorbidity scores for assessing prognosis. The relationship between schizophrenia and medical conditions is complex and challenging. With a 13-year longitudinal design of follow-up, this study allows for accurate, long-term assessment of mortality risk as well as its specific factors in patients with schizophrenia, particularly assessment of follow-up occurrence of subsequent dyslipidemia after the diagnosis of schizophrenia. This study has also applied proper statistical strategies to give a deeper insight into different clinical psychiatric circumstances that schizophrenia patients are at a risk of dyslipidemia.

Our findings suggest that specific patient-dependent risk and protective factors have influences on mortality and survival. Age, comorbidity, duration of time to subsequent dyslipidemia occurrence in schizophrenia, and use of statins were factors for mortality. Mortality tends to increase progressively in older adults with age. Mortality was also rise in patients with an age less than 50. Assessing the age-specific mortality facilitates the evaluation of possible under-reported deaths at certain ages, and is helpful for clinical intervention planning or monitoring.

Our results suggest that patients with schizophrenia and subsequent dyslipidemia had higher mortality. Mortality is also higher in elderly patients. Our findings are similar to a previous study [27], which demonstrated a significantly higher prevalence of dyslipidemia in patients with schizophrenia. The 10-year survival rate of our finding is 90%, which is lower than 94% of the 10-year cumulative survival probability reported by Healy et al. [28]. This finding may also be a reflection of earlier mortality in patients with schizophrenia and subsequent dyslipidemia. The standardized mortality rate (SMR) among schizophrenia and related psychoses is 2.5-fold of the general population [28].

Many studies have shown that the presence of both a mental disorder and a medical condition include a mix of factors, such as opportunity of receiving thorough clinical testing for and timely diagnoses of coexisting medical conditions [1,29,30]. Our findings suggest that a shorter duration of time to subsequent dyslipidemia after the diagnosis of schizophrenia increases the risk of mortality. Patients in the alive group show a substantially longer duration of time for subsequent dyslipidemia after schizophrenia diagnosis.

Our findings also suggest that comorbidity was pervasive. The assessment of comorbid diseases as risk factors for patients with mental disorders is an important implication for the prompt therapeutic opportunity [31]. The comorbid diseases are prevalent among older patients, as we found in our study. Our finding also suggests that hypertension was the most frequently found in this population. However, Ringen et al. [32] found that the major contributor to the increasing mortality is cardiovascular disease, which ranged from 40 to 50% in patients with schizophrenia.

Our finding probably reflects better survival of patients with the use of simvastatin and the clinical benefit of statin therapy. Use of statins in patients with schizophrenia and subsequent dyslipidemia has a protective role in reducing mortality rate. Particularly, simvastatin use has a protective effect on risk of death. Patients with concurrent schizophrenia and subsequent dyslipidemia have better survival odds with simvastatin treatment than without. The putative mechanism of the beneficial effects of statins might be, in part, due to their capability to modulate lipid metabolism by reducing cholesterol biosynthesis via inhibition of HMG-CoA reductase in liver, and decreasing the low-density lipoprotein (LDL) cholesterol [33]. These are particularly pertinent to late-life schizophrenia, including clinical issues relevant to survival rate and comorbidity in older patients.

Given that schizophrenia tends to become chronic, the risk of dyslipidemia becomes an important concern for patients treated with long-term antipsychotic medications. Treatment with antipsychotic medication increases susceptibility to dyslipidemia, increases both total and LDL-cholesterol levels [34,35], triglyceride [35,36], risk of dyslipidemia [27], and metabolic disturbances [10,21] in schizophrenia. Thus, treatment at early time can attenuate progression to metabolic syndrome, which is essential to survivability of patients [37], particularly for the elderly patients. However, long-acting injection use is associated with an approximately 30% lower risk of death compared with the oral dosage forms of the same medication [38]. The lowest mortality is associated with the use of second generation long-acting injection.

Identifying and screening lipid profiles regularly is important in clinical risk assessment for diagnoses of early stage of subsequent dyslipidemia. However, an earlier study indicated that up to 90% of patients do not have lipid panel collected at both baseline and follow-up time [39]. In addition, studies have shown the finding that the level of serum total cholesterol was 20 mg/dl higher in the group of schizophrenia as compared to other psychoses [3]. A longitudinal change in cholesterol levels positively associated with changes in cognitive functioning [19]. Patients are often not aware of their physical health problems, which may be a consequence of cognitive deficits associated with their mental illness.

Evidence indicates that patients with schizophrenia are more likely to experience inadequate healthcare, receive fewer assessment for medical and physical conditions, and inadequate treatment as compared to the general population [8,21]. Providing good quality care and carrying out comprehensive, systematic assessment for patients with mental illnesses is a core clinical proficiency and a fundamental and integral part of comprehensive management. The finding of this study emphasized on early intervention to patients’ needs and provided data-driven insights to improve health outcomes. A dedicated intervention should include care assessment, planning, intervention, and evaluation of risk minimization for patients with schizophrenia treated with antipsychotics in routine care. In the integrated clinical intervention, all communication, education, and treatment should be individualized to accommodate the patient’s needs [40]. It is also important that patients/families should be provided, with the most relevant information regarding their disease status based on their individual needs at different stages of the disease.

Mental health professionals can also play a critical role in improving treatment outcomes and patients’ quality of life by providing education and psychological support to patients and their family. A patient education program can help patients reduce their disease severity and improve their quality of life by engaging patients in self-management, and adherence to the treatment.

Improvements in routine health screening and monitoring for subsequent dyslipidemia in patients with schizophrenia are necessary [23]. On-the-job training programs are certainly the ways to address these challenges. The monitoring care plan for patients on second-generation antipsychotics is also of importance. Patients with elevated blood lipid levels (total cholesterol, lipoproteins, triglycerides) should seek medical care promptly. Thus, it is important to explore the possibility of using the clinical biochemical data of blood lipids as the trait marker when performing the professional mental evaluation and care.

Although the basic causes and mechanisms of mortality in patients with subsequent dyslipidemia and schizophrenia are not clear, the correlation between mortality risk due to heart disease and duration of exposure to subsequent dyslipidemia in schizophrenia remains a significant factor. To delay exposure to subsequent dyslipidemia in schizophrenia to reduce risk of developing heart diseases, mental health professionals should also consider lifestyle interventions and management in this population [6,22]. Lifestyle management includes proper exercise, early diagnosis and proper medication for dyslipidemia. Development of a cholesterol and nutritional counselling and health promotion program is important as well. The content of the program should include the cholesterol and nutritional consultation, food choices, and health promotion program as an intervention mode to patients. Mental health professionals should focus on examining patients’ unhealthy lifestyles (obesity, inactivity, alcoholism, etc.) and improving their health status by self-management education and support throughout all subsequent evaluations. However, more efforts are required to achieve a desired outcome for people with mental health difficulties than in general population.

The importance of early detection and diagnosis of dyslipidemia needs to be recognized at the organizational level by implementation of disease-specific procedures and protocols. For example, the fluctuating nature of plasma lipid levels is required to be more closely monitored. This is particularly important for patients with schizophrenia, whose changes in cognitive functioning are already difficult to assess, not knowing changes in their plasma lipid levels making care management of these patients an even bigger challenge. In this regard, routine clinical assessment of plasma lipid profile should be adopted as an important step to improve detection. Since dyslipidemia may represent a warning sign of the onset or exacerbation of a serious physical illness, failure to diagnose dyslipidemia and treatment early can bring on serious adverse repercussion. Hence, developing a strategy to implement early dyslipidemia screening and appropriate management targeting specifically on this population should be an important approach to improve their quality of life.

The main strength of this study originates from the large and comprehensive national clinical database and records we used for assessment of quality of health data, encompassing inpatient and outpatient clinical encounters. In this study, we were able to detect a difference in outcomes of deceased and alive groups, and provide an important insight into the correlates of subsequent dyslipidemia and mortality after the diagnosis of schizophrenia. However, there are some limitations. We did not have data regarding some individual clinical information, such as body mass index, alcohol intake, smoking, medication adherence, the actual lipid biochemistry data (e.g., total cholesterol concentrations), and unhealthy habits for statistical adjustment. Also, we did not retrieve data regarding the dosage of antipsychotic regimen and agents that might increase the development of dyslipidemia.

## 5. Conclusions

Results in this study show that early occurrence of dyslipidemia is associated with an increase in risk of overall mortality in patients with schizophrenia, particularly in late-life. This study shows an age-specific U-shaped mortality pattern. After 50, the risk of death increases with an increase in age. Before 50, risk of mortality increases with a decrease in age. More than two-fifths of patients died after 15 years of follow-up. Comorbid disease, such as heart failure tended to occur early among schizophrenia patients with subsequent dyslipidemia. This study has shown a significant reduction of mortality with uses of cholesterol-lowering drugs. These findings also imply that timing of detection of medical conditions influences overall survival and survivorship.

An increased integration of care through collaboration between internal medicine and psychiatry is strongly recommended. Recommendations also include lower dose antipsychotic medications, attention to which antipsychotics are used and staying away from those are particularly likely to cause weight gain and lipid disturbance, avoiding polypharmacy, and prompt referral to internal medicine.

Mental health professionals should play a pivotal role in the management of care for patients with schizophrenia. Professionals can address a wide range of aspects, such as disease assessment, and emotional support in the structured patient education. A strong relationship between patients and professionals can enhance patients’ willingness to engage in self-management in their own care. The effectiveness of a nurse-led intervention is that nurses who care for patients in an inpatient psychiatric ward are generally knowledgeable and skilled in performing metabolic monitoring. To further improve the level of care management, mental health professionals are recommended to undertake quality improvement initiative as well as on-the-job training.

## Figures and Tables

**Table 1:** Baseline demographics and clinical characteristics of the study groups.

		Deceased Group *N* (%)	Alive Group *N* (%)	*p*
Number of patients (*N* = 20,964)		2720 (12.97%)	18,244 (87.03%)	
Age (y) ^a^		37.04 (12.51)	37.55 (12.51)	0.049
Age group				
<50 yr	18–29 yr (1)	814 (29.93)	5389 (29.54)	0.027
	30–39 yr (2)	930 (34.19)	5779 (31.68)	
	40–49 yr (3)	588 (21.62)	4076 (22.34)	
50–59 yr (4)		255 (9.38)	1937 (10.62)	
60–69 yr (5)		89 (3.27)	698 (3.83)	
≥70 yr (6)		44 (1.62)	365 (2.0)	
Gender				
Female		1265 (46.51)	8471 (46.43)	0.941
Male		1455 (53.49)	9773 (53.57)	
Outcome				
Overall observation person-year ^a^		8.32 (3.34)	11.13 (2.96)	<0.0001
Months of dyslipidemia diagnosis after the diagnosis of schizophrenia ^a^		43.19 (33.75)	63.55 (41.09)	<0.0001
Major coexisting diseases				
Diabetes		526 (19.34)	1587 (8.7)	<0.0001
Hypertension		842 (30.96)	2665 (14.61)	<0.0001
Acute coronary syndrome		453 (16.65)	1094 (6.0)	<0.0001
Cerebrovascular accident		150 (5.51)	232 (1.27)	<0.0001
Heart failure		194 (7.13)	480 (2.63)	<0.0001
Alcohol-related disease		153 (5.63)	250 (1.37)	<0.0001
Liver disease (cirrhosis &failure)		90 (3.31)	138 (0.76)	<0.0001
Renal failure		583 (21.43)	2055 (11.26)	<0.0001
Chronic Obstructive Pulmonary Disease		107 (3.93)	219 (1.20)	<0.0001
Pancreatitis		527 (19.38)	2591 (14.2)	<0.0001
Malignant tumor		526 (19.34)	1587 (8.7)	<0.0001
Statin used		2720 (100.0)	18,244 (100.0)	-
Simvastatin		461 (16.95)	4453 (24.41)	<0.0001
Lovastatin		1318 (48.46)	7549 (41.38)	<0.0001
Atorvastatin		707 (25.99)	4086 (22.4)	<0.0001
Fluvastatin		234 (8.6)	2156 (11.82)	<0.0001

^a^ Values are shown as mean (standard deviation).

**Table 2:** Simple cox proportional hazard regression models for overall mortality.

		Simple Cox Model					
		Estimate	StdErr	ChiSq	HR	95% CI	*p*
**Age Group**	2 vs. 1	0.0655	0.0480	1.8599	1.07	(0.97–1.17)	0.172
	3 vs. 1	0.0284	0.0541	0.2757	1.03	(0.93–1.14)	0.599
	4 vs. 1	0.0202	0.0718	0.0789	1.02	(0.89–1.17)	0.778
	5 vs. 1	−0.0769	0.0935	0.6756	0.93	(0.77–1.11)	0.411
**Sex**	M vs. F ^a^	−0.0382	0.0384	0.9884	0.96	(0.89–1.04)	0.320
**Statin used**							
Simvastatin	1 vs. 0 ^b^	−0.3079	0.0512	36.1885	0.74	(0.66–0.81)	<0.0001
Lovastatin		0.1664	0.0384	18.7393	1.18	(1.10–1.27)	<0.0001
Atorvastatin		0.1238	0.0437	8.0199	1.13	(1.04–1.23)	0.005
Fluvastatin		−0.1951	0.0684	8.1284	0.82	(0.72–0.94)	0.004
**Months of dyslipidemia diagnosis after schizophrenia**		−0.0159	0.0006	837.0958	0.98	(0.98–0.99)	<0.0001
**Comorbidities**							
Diabetes	1 vs. 0 ^b^	0.8458	0.0486	303.3864	2.33	(2.12–2.56)	<0.0001
Hypertension		0.8275	0.0415	397.9726	2.29	(2.11–2.48)	<0.0001
Acute coronary syndrome		0.9680	0.0515	353.6648	2.63	(2.38–2.91)	<0.0001
Heart failure		1.3872	0.0841	272.3742	4.0	(3.40–4.72)	<0.0001
Alcohol-related disease		1.0662	0.0746	204.5353	2.9	(2.51–3.36)	<0.0001
Liver disease (cirrhosis, failure)		1.3065	0.0832	246.3716	3.69	(3.14–4.35)	<0.0001
Renal failure		1.4277	0.1072	177.2499	4.17	(3.38–5.14)	<0.0001
Chronic Obstructive Pulmonary Disease		0.6895	0.0467	217.6634	1.99	(1.82–2.18)	<0.0001
Pancreatitis		1.1462	0.0987	134.9501	3.15	(2.59–3.82)	<0.0001
Malignant tumor		0.4006	0.0486	68.0573	1.49	(1.36–1.64)	<0.0001

^a^ M vs. F, male vs. female; ^b^ 1 vs. 0, treated vs. non-treated.

**Table 3:** Multivariate-adjusted cox proportional hazard regression models for overall mortality.

		Multiple Cox Model					
		Estimate	StdErr	ChiSq	HR	95% CI	*p*
Age Group	2 vs. 1	0.0777	0.0481	2.6178	1.08	(0.98–1.19)	0.105
	3 vs. 1	0.0250	0.0543	0.2116	1.03	(0.92–1.14)	0.645
	4 vs. 1	0.0263	0.0722	0.1325	1.03	(0.89–1.18)	0.715
	5 vs. 1	−0.0280	0.0938	0.0893	0.97	(0.81–1.17)	0.765
Sex	M vs. F ^a^	−0.0530	0.0387	1.8749	0.95	(0.88–1.02)	0.170
**Statin used**							
Simvastatin	1 vs. 0 ^b^	−0.1159	0.0571	4.1127	0.89	(0.80–1.00)	0.042
Lovastatin		−0.0231	0.0429	0.2906	0.98	(0.90–1.06)	0.589
Months of dyslipidemia diagnosis After onset of schizophrenia		−0.0126	0.0006	484.0781	0.99	(0.99–0.99)	<0.0001
**Comorbidities**							
Diabetes	1 vs. 0 ^b^	0.3145	0.0509	38.1162	1.37	(1.24–1.51)	<0.0001
Hypertension		0.2904	0.0462	39.4443	1.34	(1.22–1.46)	<0.0001
Acute coronary syndrome		0.2905	0.0583	24.8708	1.34	(1.19–1.50)	<0.0001
Heart failure		0.6103	0.0903	45.6872	1.84	(1.54–2.20)	<0.0001
Alcohol-related disease		0.7503	0.0815	84.7271	2.12	(1.81–2.49)	<0.0001
Liver disease (cirrhosis & failure)		0.5204	0.0924	31.6992	1.68	(1.40–2.02)	<0.0001
Renal failure		0.7805	0.1099	50.4175	2.18	(1.76–2.71)	<0.0001
Chronic Obstructive Pulmonary Disease		0.3501	0.0491	50.8535	1.42	(1.29–1.56)	<0.0001
Pancreatitis		0.5256	0.1052	24.9487	1.69	(1.38–2.08)	<0.0001
Malignant tumor		0.1476	0.0494	8.9264	1.16	(1.05–1.28)	0.002

^a^ M vs. F, male vs. female; ^b^ 1 vs. 0, treated vs. non-treated.

## Data Availability

Data are available from the National Health Insurance (NHI) research database published by the Taiwan NHI administration. Due to the legal restrictions imposed by the government of Taiwan concerning the Personal Information Protection Act, and related regulations, the data cannot be made publicly available.
